# Are Neurodynamic Organizations A Fundamental Property of Teamwork?

**DOI:** 10.3389/fpsyg.2017.00644

**Published:** 2017-05-02

**Authors:** Ronald H. Stevens, Trysha L. Galloway

**Affiliations:** ^1^Brain Research Institute, UCLA School of MedicineCulver City, CA., USA; ^2^The Learning Chameleon, Inc.Culver City, CA, USA

**Keywords:** teamwork, EEG, social coordination, team neurodynamics, information theory, entropy, uncertainty

## Abstract

When performing a task it is important for teams to optimize their strategies and actions to maximize value and avoid the cost of surprise. The decisions teams make sometimes have unintended consequences and they must then reorganize their thinking, roles and/or configuration into corrective structures more appropriate for the situation. In this study we ask: What are the neurodynamic properties of these reorganizations and how do they relate to the moment-by-moment, and longer, performance-outcomes of teams?. We describe an information-organization approach for detecting and quantitating the fluctuating neurodynamic organizations in teams. Neurodynamic organization is the propensity of team members to enter into prolonged (minutes) metastable neurodynamic relationships as they encounter and resolve disturbances to their normal rhythms. Team neurodynamic organizations were detected and modeled by transforming the physical units of each team member's EEG power levels into Shannon entropy-derived information units about the team's organization and synchronization. Entropy is a measure of the variability or uncertainty of information in a data stream. This physical unit to information unit transformation bridges micro level social coordination events with macro level expert observations of team behavior allowing multimodal comparisons across the neural, cognitive and behavioral time scales of teamwork. The measures included the entropy of each team member's data stream, the overall team entropy and the mutual information between dyad pairs of the team. Mutual information can be thought of as periods related to team member synchrony. Comparisons between individual entropy and mutual information levels for the dyad combinations of three-person teams provided quantitative estimates of the proportion of a person's neurodynamic organizations that represented periods of synchrony with other team members, which in aggregate provided measures of the overall degree of neurodynamic interactions of the team. We propose that increased neurodynamic organization occurs when a team's operating rhythm can no longer support the complexity of the task and the team needs to expend energy to re-organize into structures that better minimize the “surprise” in the environment. Consistent with this hypothesis, the frequency and magnitude of neurodynamic organizations were less in experienced military and healthcare teams than they were in more junior teams. Similar dynamical properties of neurodynamic organization were observed in models of the EEG data streams of military, healthcare and high school science teams suggesting that neurodynamic organization may be a common property of teamwork. The innovation of this study is the potential it raises for developing globally applicable quantitative models of team dynamics that will allow comparisons to be made across teams, tasks and training protocols.

## Introduction

We all exist in continual perception/action cycles where we sample the environment, actively compare our perceptions with our probabilistic representations of the incoming information, adjust our models accordingly and then resample and/or change the environment. The goal of these cycles is to optimize the values and costs of future actions in order to minimize surprise. At the intersection of value and costs is the uncertainty that becomes resolved by this process.

Much of this decision-making activity is orchestrated by implicit brain process and occurs rapidly (Hsu et al., 2005); it has been proposed that human mental processes have evolved to minimize perception-model errors across systems and avoid the costs of surprise (Barlow, 1961; Friston, 2010).

**Figure 1:**
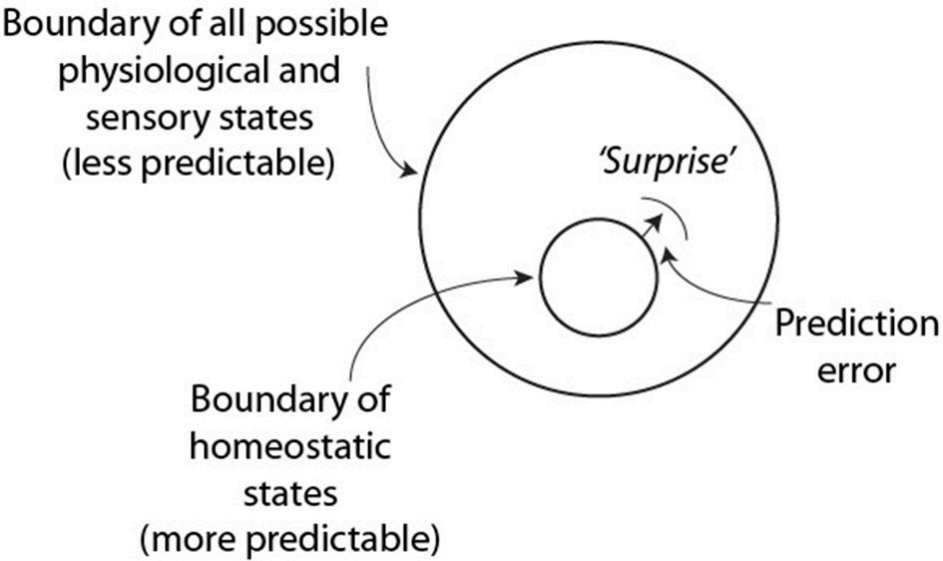
**Tenants of the free energy principle and the predictability of surprise** (based on Friston, 2010). This figure shows a part of the homeostatic boundary that became shifted by a surprising event in the environment.

These ideas have been encapsulated by Friston (2010) into a model, the free energy principle that develops a unified account of perception, action and learning (Figure 1). The free energy principle proposes that of the large number of physiological and sensory states that exist, there is a high probability that an individual's current state exists within a much smaller state space roughly defined by homeostatic requirements; i.e., the system is optimized and predictable for the most part. Occasionally however, large prediction errors arise between incoming information and internal probabilistic representations and these errors trigger parts of these systems to drift from homeostatic boundaries and the system becomes less predictable as a result of this surprise. From information theory, this change in predictability can be described as an increase in the uncertainty, or entropy of the system (abbreviated *H* in this paper).

Entropy is the average surprise of outcomes sampled from a probability distribution or density. A density with low entropy means that, on average, the outcome is relatively predictable, while a system with higher entropy would be less predictable. Entropy is therefore a measure of uncertainty.

When entropy gets too high new cognitive organizations are thought to emerge (Zipf, 1949), and through general error correcting and learning processes the system returns to within the homeostatic bounds. It is these reorganizations that we are interested in, primarily at the neurodynamic level of teams.

It is not difficult to extrapolate the free-energy principle to teams as surprises also happen during teamwork, especially with teams performing complex tasks where no two task instances are the same. In teams however, each person must now consider their actions, not only with regard to their roles in a changing environment, but also with regard to those other persons, each of whom is a complex system with a slightly different dynamic perspective of the environment. Nevertheless, the overall idea of minimizing the prediction error between incoming information (from the task and other team members) and an individual team members' representation of the situation is analogous to minimizing surprise.

As resolving the cross-person (i.e., cross-brain) uncertainty will occur external to individual brains (through speech or gestures for instance), the mechanisms for optimizing the prediction error in teams are likely to be more complex and lengthy than those postulated to exist in individuals. Occasionally, due to these complexities and temporal delays, a team's decisions will be suboptimal and the team must dynamically reorganize into a configuration that is more appropriate for the immediate situation or alternatively, change the situation. This requires not only a re-assessment of the present situation, but also the mental “playing forward” of alternative approaches, with the eventual selection of an action by the team with potentially the best outcome (Schacter et al., 2007).

In this study we ask: What are the neurodynamic properties of these reorganizations in teams, how are they induced, and how do the dynamics differ among the team members? We take an information-organization approach in answering these questions in this paper as we believe this may provide a general and extensible quantitative framework for investigating teamwork across different teams performing different tasks.

The paper begins with an illustration of the overall modeling approach using the hypothetical dynamics of a theoretically perfect team where we speculate on how these dynamics might change when team members get “out of synch.” This section is followed by more detailed descriptions of the modeling approach for exploring the neurodynamical and informational relationships between the organizations of individuals and teams. The third section provides empirical evidence for the variety and importance of different neurodynamic organizations during teamwork. These sections draw from studies we have performed with high school teams performing map navigation tasks (Stevens and Galloway, 2014), submarine teams performing required navigation training exercises (Stevens et al., 2011, 2013; Stevens and Galloway, 2015) and healthcare teams (Stevens et al., 2016a). The similar dynamical and observational principles arising from these different tasks suggest that the phenomena being studied might be a fundamental property of teamwork.

## Materials and methods

### Neurodynamics of a “theoretically” perfect team

The goal of the first section is to describe how the constraints of inter-personal communication and joint resolution of uncertainty might contribute to the changing neurodynamics of teams performing complex tasks. This example focuses on a three-person team although previous data from submarine navigation teams suggests the approach can be scaled to 5–6 person teams with certain simplifying assumptions that will be discussed in subsequent sections.

A starting assumption behind this example is that the efficiency and effectiveness of a team performing a complex task is enhanced by the fast and precise sharing of information, regardless of the interoceptive or exteroceptive uncertainty or noise in the system. We begin by postulating that each of the three team members has three possible energy levels, below average, average and above average, which represent the EEG signal power (in micro-volts) in a frequency bin recorded from scalp sensors; these levels can change every second. These three states could easily be quarters or fifths, or other discrete bins, with the associated scale-up costs in model computation.

The one-second interval is a theoretically plausible number for teams as periods of functional brain connectivity associated with speech or playing guitar in duets (Stephens et al., 2010; Sanger et al., 2012), and non-verbal recognition (Hari, 2006) occur in the 250–500 ms time range, or a bit over a half a second for a two person action-response round trip; in reality individuals in teams probably speed this up by predicting ahead, although at a cost of increased uncertainty (Hsu et al., 2005).

The analysis we describe is simplified as only one EEG frequency bin is being modeled that is within the range of human cognition, and easily available for research “in the wild.” This dimension generally spans the 0.1 to 100 Hz frequency range as below this range other physiologic signals generated by respiration, heartbeats, electrode pops etc. may confuse the patterns and above this, electromyographic signals become a serious confounder. We also assume that the data was recorded from a single sensor site on the scalp. As described in the next section we currently model 1 Hz frequency bins from the 1- 40 Hz EEG frequency range that is simultaneously obtained from up to 19 sensor sites; i.e., the examples described below are generally repeated 760 (i.e., 40 × 19) times for each person in an experiment (or 2,280 times total for a 3-person team).

**Figure 2:**
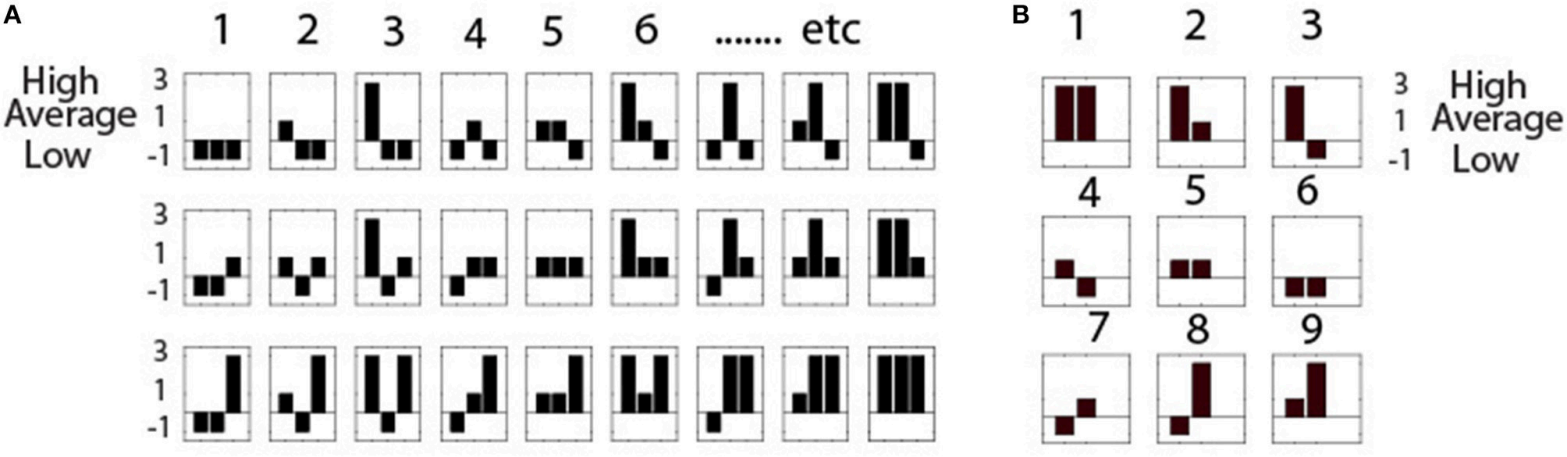
**Neurodynamic State Spaces**. **(A)** Neurodynamic symbol state space for a three person team with high, medium or low EEG power levels. The legend on the left shows that low power is represented by -1, average by 1, and above average power levels by 3. As these values are treated symbolically they do not affect calculations, but do enhance visualization. Each of the three histograms in each *NS* represents a team member. **(B)** The nine symbol *NSS* for dyads.

Next, a way of representing the state of each individual as a part of a team at any moment of the performance is needed; i.e., the state of each team member in relation to the other team members as well as to the immediate context of the task (Box 1). These combinations are represented as symbols with histograms showing the power level combinations for the team; with three energy states per person, and three persons, 27 unique symbols are needed. These symbols are termed Neurodynamic Symbols (*NS*), and the 27 symbols form a collection of states that together describe the expression of *NS* for a performance; this collection is termed a Neurodynamic State Space (*NSS*) and is shown in Figure 2A for a three-person team and Figure 2B for a dyad. A data stream of these symbols contains a neurodynamic history of the team performance, much like the codons in DNA.

Box 1 **Glossary of terms.**

- *Neurodynamic Symbols* (*NS*) are symbolic representations of the momentary EEG power levels of a neurodynamic marker for each team member.
- *Neurodynamic Symbol States (NSS)* are a collection of *NS* that together describe a team's performance.
- *Neurodynamic Data Streams (NDS)* are the second-by-second concatenated sequences of *NS* that temporally span a task performed by the team.
- *Neurodynamic Entropy (NS*_*H*_*)*, also called team entropy, is a quantitative measure of the distributions of *NS* in a *NDS* when examined over a moving window of time, often 60 s or 100 s. The quantitative information unit is called a bit, where **one** bit of information indicates that on average, the uncertainty of a process is reduced by a factor of **two** with **one** bit of information.
- *Neurodynamic Organization (ND*_Ω_*)* is a quantitative estimate of organization reflecting periods of increased neurodynamic order. *ND*_Ω_ is calculated by subtracting the Shannon entropy of the *NDS* obtained over a 60 or 100s moving window, from the entropy of the *NS* stream after it has been randomized (i.e., *ND*_Ω_ = *NS*_*H*__*random*_ - *NS*_*H*_). Neurodynamic organization can be calculated either from the entropy levels of individual team members or from the team entropy. When referring to individual's neurodynamic organization we will prefix it with the italicized word individual, i.e., *individual ND*_Ω_.

The *NSS* contain topological structures that enhance the interpretation and visualization of team neurodynamics. The symbols toward the beginning of the *NSS* in Figure 2A (i.e., 1–4) represent periods where most of the team members had low EEG power levels, while those *NS* toward the end (24–27) represent times where most team members had high EEG power. Also, moving down each column in the *NSS* shows that only one person of the team is changed, going from low to average to high power. This *NSS* serves as a lookup table when visualizing the neurodynamics of teams.

Now imagine a fully connected, tightly coupled (in a network sense) experienced team so familiar with their goals, individual tasks, and team roles that they can engage in “mental time travel” (Schacter et al., 2007) and predict the future such that the future holds few surprises. For such a team, each person's responses to changes in the task and the responses of other team members would be limited primarily by the latencies imposed by cognitive and motor systems (Suzuki et al., 2012).

This theoretically perfect team would also understand and trust their teammate's likely responses, so communication and strategizing delays would be those imposed by the mechanics of action understandings, speech processing and information exchange described earlier. To the extent that the task activities and team member interactions are sufficiently predictable to avoid surprises, the dynamical structure of this team might be highly variable as the members maximize the flows of team information content by flexibly using all of the states available in the 27 *NSS*.

This idea of maximizing variability to maximize information transmission might seem at odds with the more predictable smaller physiologic and sensory state space that was optimized for homeostatic processes in Figure 1. The difference is in the temporal scales over which processes are optimized. The constraints described in Figure 1 have been optimized by evolution to make life possible. These processes continually transfer information from the environment to the genome to match the homeostatic boundaries with the selection pressures of the environment. In teams there is no similar genetic selection during a teams' lifetime, and the team's success depends more on maximizing the efficiency and effectiveness of the major task and teamwork processes, with the transfer of information among team members being paramount.

**Figure 3:**
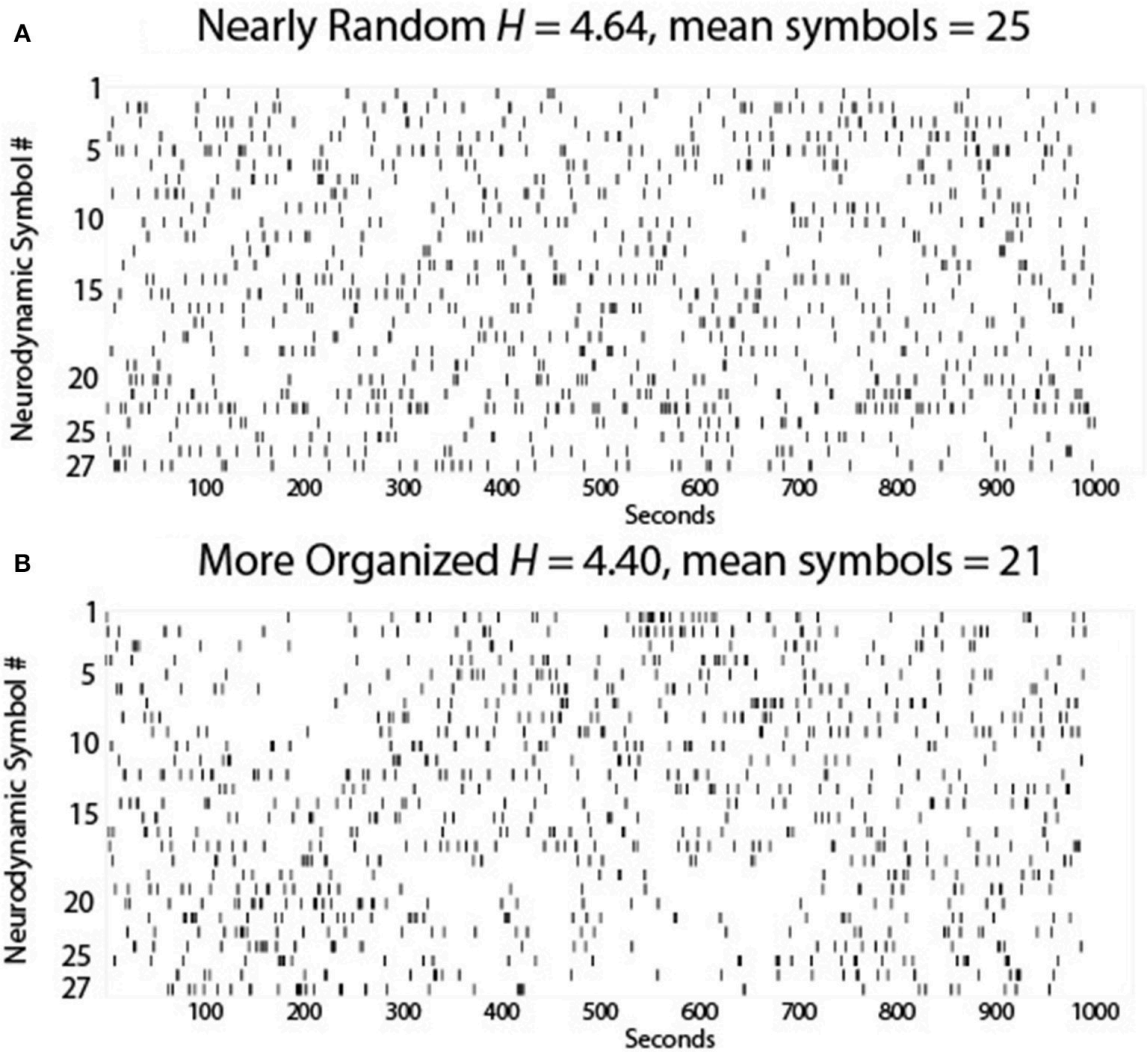
**The 27 *NS* of the *NSS* are plotted each second for (A)** a theoretically near-perfect team, and **(B)** a team characterized by less *NS* variability. The symbol number is on the *Y*-axis and the time in seconds is on the *X* axis.

An example of the possible dynamics of this team is shown in Figure 3A where each of the 27 symbols in the *NS* data stream are sequentially plotted. Here the momentary changes at the neurodynamic level associated with the task work and teamwork would be couched within the 1 s sampling window so there are few repeating symbols due to slow social coordination and information sharing.

While the symbol distribution appeared random for this team, the entropy of this Neurodynamic Symbol Data Stream (abbreviated *NDS*) (*H* = 4.64 bits) showed that it was less than the theoretical maximum entropy for 27 symbols (*H* = 4.76 bits) indicating a non-random distribution of symbols, i.e., there is some hidden organization, perhaps due to system noise or the team's threshold tolerance for surprise. Nevertheless, the overall neurodynamic variability of the team was high suggesting efficiency as a discrete symbol set with high variability can convey more information than a symbol set with low variability. These ideas are consistent with the efficient coding hypothesis (Barlow, 1961) which states that the goal of the nervous system is to maximize information about the environment, and in doing so, to minimize the energy expended for each bit of information.

Now suppose one (or more) team members was less experienced than the others and became delayed by the unfolding events leading to increased surprise (in the free-energy principle sense) and deliberation by that person and the team (Kaufman et al., 2015). To resolve this new uncertainty the team members would need to become more predictable (i.e., less variable) to each other and this higher predictability could be accomplished by increased organization. In our theoretical situation this increased neurodynamic organization would be characterized by increased *NS* redundancy. Increased redundancy of information is common in nature as it is one way to ensure effective communication.

Figure 3B shows two periods of increased *NS* redundancy in the 1000 s performance. The first occurred between ~125 and 275 s and was characterized by the selective expression *NS* 18–27, which from Figure 2 were periods where there was a tendency toward higher EEG power levels across the team members, and the second was between ~525 and 625 s where *NS* 1–10 were selectively expressed i.e., a tendency for lower EEG power across the team. During these periods the teams would be acquiring more information to reduce the individual and team's prediction errors, and bring their prediction model of the world closer the real model of the situation.

As described in the next section, the reduced variability of the symbols during these periods would result in lower entropy levels. Practically this might occur by changing the flow or content of information sharing across the team (Kiekel et al., 2004), deliberately slowing down the pace of the task (Moulton et al., 2010), re-organizing the structure of the team, or re-organizing the structure of the task.

Depending on the coupling of the systems involved in teamwork (Hasson et al., 2011), these changing organizations would ripple over time until homeostasis for the team is re-established. Depending on the situation, the team would either return to pre-perturbation entropy levels or remain in a more organized state attentive to further surprise.

While acquiring more information to reduce uncertainty is beneficial for the team, what are the costs? Team re-organizations require energy. In information theory, reducing uncertainty is synonymous with acquiring more information, and acquiring more information requires energy. According to Szilard (1929), the act of acquiring information from a system generates entropy, or equivalently, it has an energetic cost due to the very nature of the procedure. He showed that the minimum amount of energy required to determine one bit of information is *k*T ln(2) Joules/bit, a quantity Landauer (1961) generalized to any way of manipulating or processing information such as measuring, encoding, displaying, a yes/no decision, etc. From the second law of thermodynamics, as the organization of a team increases (i.e., decreased entropy), it must increase entropy somewhere else, the most likely source being through energy production where complex molecules (sugar, ATP) or macromolecules (glycogen) are broken down, increasing the disorder. Such increased energetic costs associated with social coordination have been seen as increased BOLD signals in the medial prefrontal cortex of individuals simultaneously scanned during a deception team task (Montague et al., 2002).

The above discussion raises questions: Can we begin to populate models of teamwork with quantitative data that reflects the above ideas, and with what is understood to be expertise? Are team members in fact fully connected, and if so, how tightly linked are the couplings across different team members during different teamwork measures? Are there preferred couplings among team members depending on the task, or training protocol, or training site, and does this make a difference? How closely related are the models being revealed by neurodynamics, communication and behavioral measures? The next section describes the modeling approaches that might be used to approach these questions.

### Tasks and participants

#### Map navigation task

In the Map Task (MT) the team members faced each other while viewing a computer displaying a map with multiple landmarks (Doherty-Sneddon et al., 1997). The two maps were similar but not identical and students could not see each other's map. The instruction giver [Giver, abbreviated (G)], had a printed path through the landmarks and verbally guided the follower [Follower, abbreviated (F)] in duplicating that path. Students completed the Map Task using speech exchanges to determine where the paths should be drawn. The resulting speech was unscripted, fluent and contained easily identified goals (Stevens and Galloway, 2014).

#### Submarine piloting and navigation

Submarine Piloting and Navigation (SPAN) simulations were required exercises for Junior Officers in the Submarine Officer Advanced Candidacy course at the US Navy Submarine School. SPAN sessions contained three training segments: Briefing; Scenario; and Debriefing. Briefing was where the team reviewed the environmental conditions and other ships in the area, and statically established the submarine's position. The Scenario was the training part of the navigation simulation where events included: encounters with approaching ships, the need to avoid shoals, changing weather conditions, and instrument failure. The Debriefing was an after-action review where all team members participated in critical performance discussions (Stevens et al., 2012).

#### Healthcare simulations

The simulations developed for healthcare also followed the standard training format beginning with a Briefing describing the goals of the exercise. This was followed by a short 5–10 min introduction including the simulated patient history which set the stage for the task simulation that lasted 15–20 min. A reflective Debriefing was then led by the instructor (15–20 min). The core construct of this simulation series was ventilation with procedural goals of demonstrating (1) the technical skills of supporting the airway of an obtunded patient, (2) the cognitive goals of carrying out team-based approaches to patients with decreased mental status; and, (3) practicing role assignment during care of a patient with an urgent/emergent clinical condition (Stevens et al., 2016b).

#### Ethics statement

Informed consent protocols were approved by the Biomedical IRB, San Diego, CA, the OSF Healthcare Institutional Review Board, and the Naval Submarine Medical Research Laboratory Institutional Review Board, and written informed consent was signed by all participants to participate in the study and to have their images and speech made available for additional analysis. To maintain confidentially, each subject was assigned a unique number, known only to the investigators of the study and subject identities were not shared. This design complies with DHHS: protected human subject 45 CFR 46; FDA: informed consent 21 CFR 50. The selected examples presented in this paper were chosen from 15 Map Task, 16 Submarine Piloting and Navigation, and 6 healthcare team performances.

### Electroencephalography

Prior to neurodynamically modeling the team the raw electroencephalographic (EEG) data from each team member were synchronized with each other through markers inserted into the data streams during data collection and then visually inspected for motion and other artifacts. Bad sensor channels or components identified as being enriched for eye blinks or heartbeats were discarded as described below.

EEG data was collected using the Quick 20 EEG headset from Cognionics, Inc. (Carlsbad, CA), with sensor locations at F7, Fp1, Fp2, F8, F3, Fz, F4, C3, Cz, P8, P7, Pz, P4, T3, P3, O1, O2, C4, T4 in a monopolar configuration referenced to linked earlobes. EEG data were preprocessed for each team member using FieldTrip (Oostenveld et al., 2011) by applying high-pass (0.5 Hz) and low-pass filters (50 Hz) and removing bad channels (max = 2). Spatially transformed independent component analysis was performed with RUNICA (Delorme et al., 2012) to detect and remove artifacts associated with eye blinks, electrocardiogram and electromyogram activity. Following artifact rejection using RUNICA, data were back-reconstructed and the channels removed prior to RUNICA decomposition were interpolated back into the data by spherical interpolation. Frequency decomposition was performed by first segmenting data into 1 s epochs. The data were then windowed using Hanning taper and the frequency content of each trial was measured at 1 Hz intervals from 1 to 40 Hz using Fast Fourier Transform.

### Team neurodynamic modeling

The goal of team neurodynamic modeling is to develop data streams that contain temporal information about the organization, function and performance of teams. In this study we highlight the 10 Hz frequency which is involved in attention and prioritizing stimuli (Klimesch et al., 2007; Klimesch, 2012), the 16 Hz frequency that is involved in action understandings (Hari, 2006), and the 40 Hz frequency involved in maintaining working memory and long-term memory encoding and retrieval (Roux and Uhlhaas, 2014; Bonnefond and Jensen, 2015). These frequencies were chosen based on prior work that revealed that these frequency bands had particular relevance for team neurodynamics (Stevens and Galloway, 2014, 2015).

**Figure 4:**
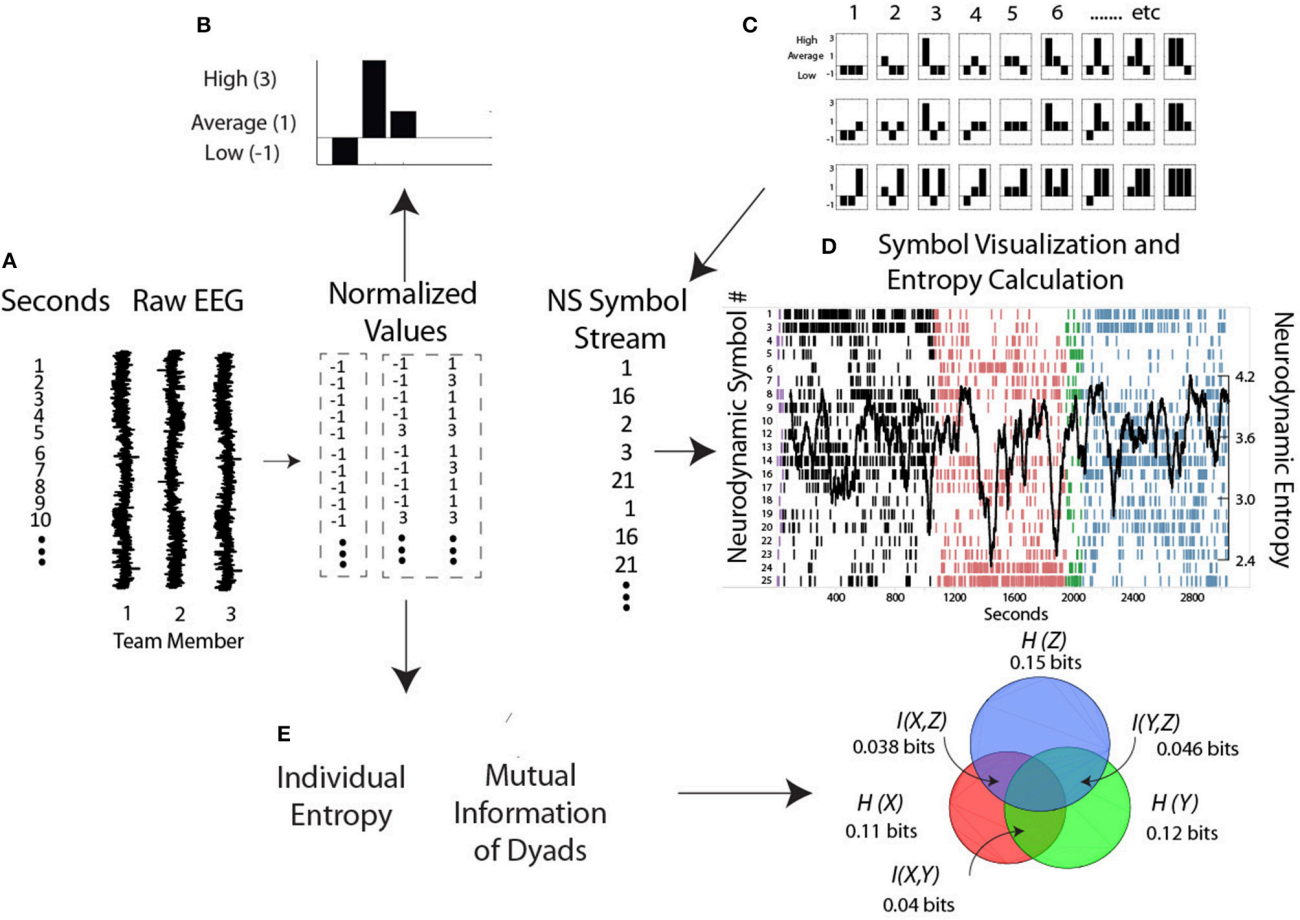
**Steps for generating and modeling neurodynamic data streams**. **(A,B)** The raw EEG signals from each person are discretized each second into low, average, and high power levels and assembled into a *NS*. **(C)** The symbol matching the three person power vector is determined from the *NSS* lookup table and assembled into a *NDS*, where, **(D)** the symbols are visually mapped and a moving average of entropy calculated each second. **(E)** Levels of Individual Entropy and the Mutual Information of dyad pairs are calculated from the normalized symbols and used for subsequent team modeling as described in the text.

As described earlier, the initial modeling step is to generate the power level vectors (i.e., −1, 1, and 3's) from the raw EEG data from each person, and create the *NS* for each second of the performance (Figure 4). The normalized power vector was presented to a previously trained artificial neural network and matching *NS* were assembled into a *NDS* which was updated each second with a new symbol (Stevens and Galloway, 2014). The structure (i.e., information) in these data streams was visualized by plotting the symbol expressed each second. By classifying the set of symbols over entire performances containing different segments (i.e., Briefing, Scenario, and Debriefing segments shown by the different colors) the neurodynamic models generated encompasses a comprehensive set of task situations/loads (Fishel et al., 2007).

According to information theory, a data stream with 27 symbols has a theoretical entropy level of 4.76 bits if the symbols were equally distributed (i.e., a uniform distribution), and so if we observe a data stream to have an entropy of 4.58 bits, then we know that there is a “hidden” structure in that data, i.e., some symbols are expressed more frequently than others. But this difference does not tell us where the structure is, it only tells by how much. For that information the performance needs dividing into smaller time units like the Briefing, Scenario and Debriefing task segments or over even smaller time windows within these segments, i.e., an entropy rate. So if we determine the entropy over a 60 s or 100 s length segment and the entropy level is now 4.08 bits instead of 4.58 bits the new information we have gained is equal to the difference in *H* before and after we received that information, i.e., 0.5 bits. While it does not matter for the aggregated *H* levels, for practical teamwork purposes we need to know what symbols are lost, what symbols remain, and how they are distributed in the data stream, it is not sufficient to know just that some symbols remain or are gone.

Figure 4D is a plot of the 39 Hz (gamma) frequency bin from a healthcare team and shows several important features. In the Briefing (black) and Debriefing (blue) task segments the dominant symbols expressed were *NS* 1 and 2 representing times when most team members had low EEG gamma power. In contrast, the dominant symbols in the Scenario (pink) were *NS* 26 and 27 indicating times when most team members had high EEG gamma power.

It is important to note that high or low EEG power in the frequency bands is not necessarily good or bad, as different power levels serve different purposes; for example during spontaneous coordination the mu medial rhythm is synchronized (i.e., high power), but becomes suppressed or desynchronized (i.e., low power) during social interaction (Tognoli and Kelso, 2015). Similarly, synchronized (i.e., high power) alpha may provide a mechanism for selective attention while desynchronized alpha may promote working memory formation (Klimesch, 2012). It is also important to note that from a neurodynamic organization perspective, preferential expression of symbols representing high power or low power will show equivalent entropy levels if the variability of the symbols is the same. This is also shown in Figure 4D as large NS entropy decreases occurred when the gamma levels were either low (Briefing and Debriefing) or high (Scenario) across the team. Large entropy fluctuations identify performance periods warranting additional study through video and audio analysis, or semantic structure analysis.

### Individual entropy and mutual information

The next calculated variable is Individual Entropy (*IE*) (Figure 4E) where the normalized EEG values of each person are treated symbolically and then Shannon's entropy is calculated over a moving window as described above. It is not clear what Individual Entropy represents, although it can be thought of as the neurodynamic organizations of individuals as they perform their taskwork as well as their teamwork.

Short and long-term changes in *NS*_*H*_ identify fluctuating periods of team neurodynamic organization but they provide little information about possible neurodynamic synchronization among the team members and the possible roles of these interactions during teamwork; mutual information descriptions help supply this data. Mutual information (*MI*) is a measure of the mutual dependence of two variables, or how much knowing the value of one variable decreases the uncertainty of the value of the other. Mutual information was originally described in noisy channel communication as the information in the output channel that was present in the input channel, and has been widely used for evaluating information representations, transmissions, and content in single neurons and populations of neurons in stimulus- responses paradigms (Schneidman et al., 2003; Onken et al., 2014). We use *MI* to determine the amount of shared information between two team members, periods which we cautiously refer to as times of synchrony (Stevens and Galloway, 2016). Currently it is not known what the remaining information is after subtracting the *MI*. Possibilities include it being noise, or perhaps information more closely related to an individual's task work rather than teamwork.

**Figure 5:**
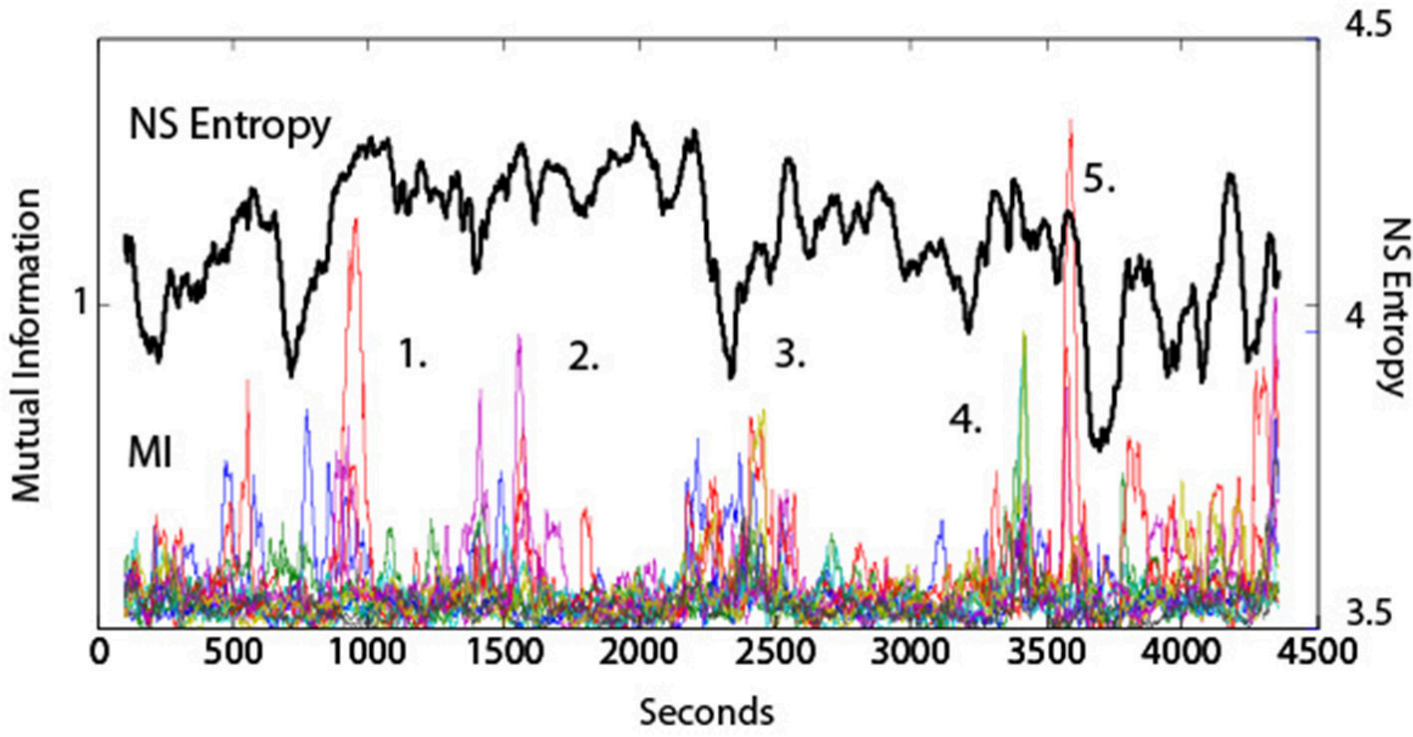
**Dynamical comparisons of *NS*_*H*_ and *MI***. The *NS*_*H*_ was averaged across all frequencies and all sensors, and the *MI* values were averaged across the fourteen dyad pairs shown by the colored lines. The numbers represent performance events surrounding those time periods. (1) The team was having difficulty remembering the sequence of buoys to use when establishing the ship's position. (2) The team was preparing for a turn into difficult waters with other ship traffic. (3) A simulation “Pause” was called by the Assistant Navigator to express his concerns with the team. (4) A Man Overboard event. (5) Beginning of the Debriefing segment.

The symbols used for calculating the *MI* of dyads were the same as for *IE* i.e., the normalized EEG vectors (−1, 1, and 3), and in all studies a moving average window approach for *MI* data reporting was used as described above for *NS*_*H*_. An example of the relationships between *MI* and team entropy is shown in Figure 5 for a submarine navigation team composed of six team members. In this figure there are five major events marked that were regarded as significant by the instructor. The individual colored lines in the *MI* plot represent the fourteen different dyad combinations of the team. The periods of elevated *MI* contained many of the dyad combinations suggesting that periods of synchrony are not always present, but when they are they involve many of the team members. The correlation between *MI* and *NS*_*H*_ was low (*r* = 0.02) at a time lag of zero indicating that while *MI* may be near periods of decreased *NS*_*H*_, they may not always be the same periods.

## Results

### Quantitative models of team member organization during teamwork

This section provides examples describing how the different information flows in the neurodynamic data streams can be used to quantitatively:

1. Determine the degree of team synchrony as defined by mutual information.
2. Determine the contributions made by the individual team members to the overall team's neurodynamic organizations; and,
3. Dissect the momentary neurodynamics of individual team members to determine how these dynamics relate to the overall dynamics of the team and the task.

The studies in this section integrate *NS*_*H*_ dynamics, *IE* dynamics and *MI*, and introduce related information measures which are joint entropy (*JE*) and conditional entropy (*CE*). As illustrated in Box 2, *JE* is the sum of the *IE* of each team member, and *CE* is the entropy remaining after the *MI* between two persons is removed (shown in gray).

Box 2 **Information measures**

**Figure:**
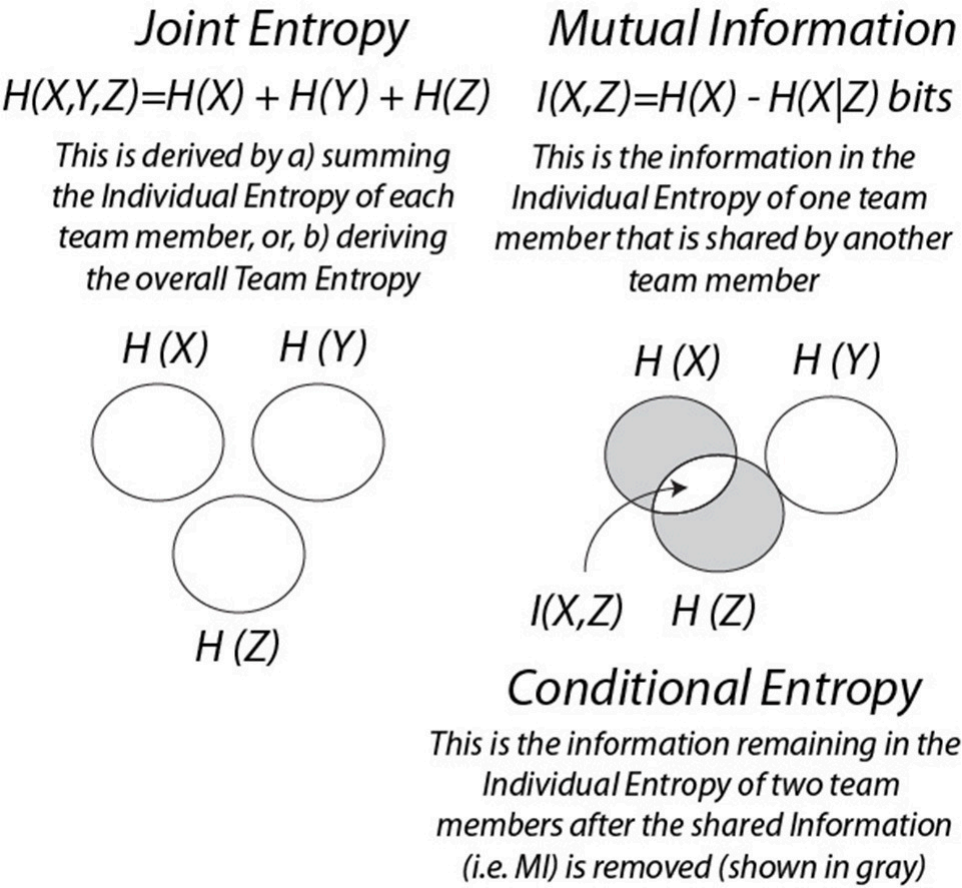

**Figure 6:**
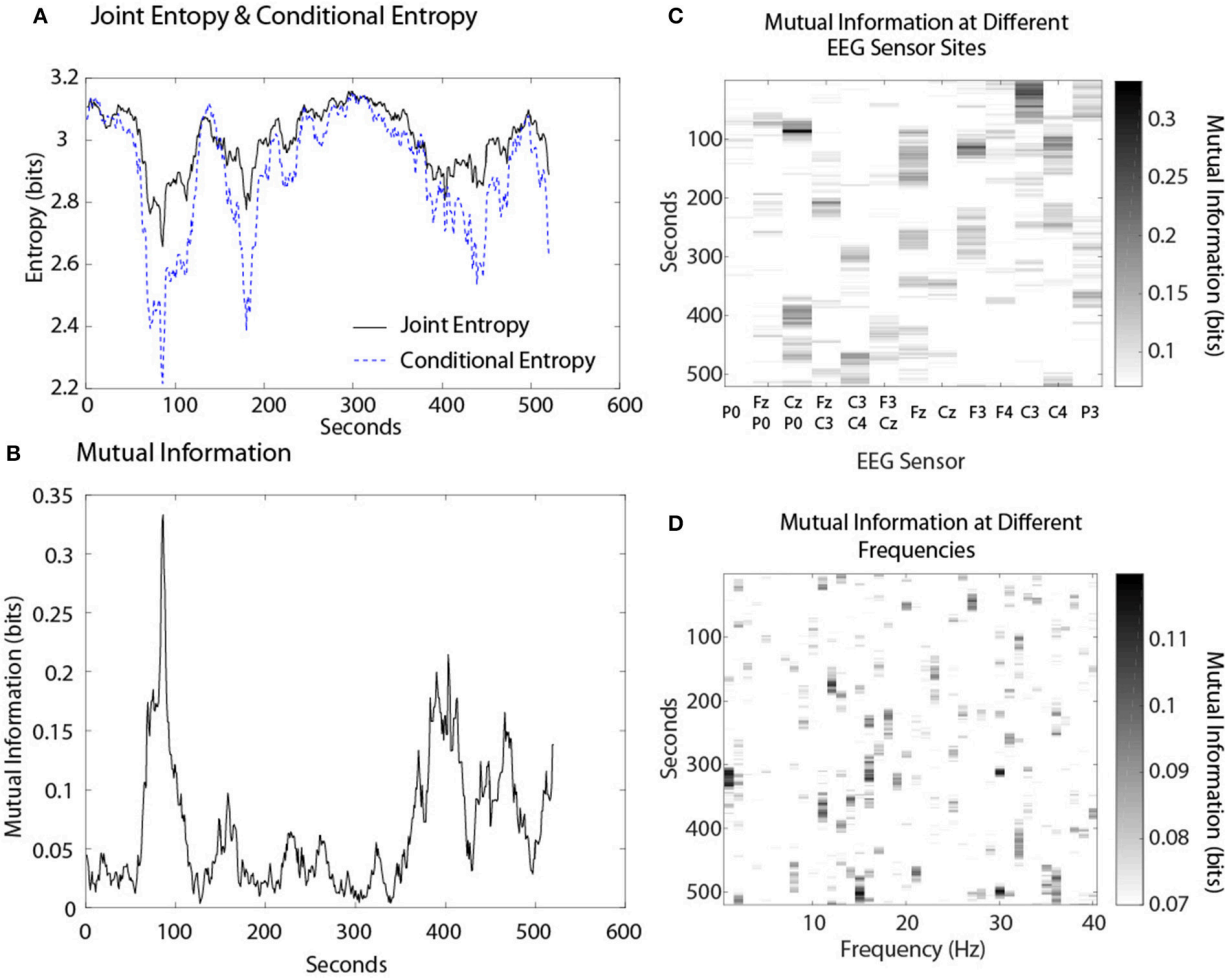
**(A)** Dynamics of joint entropy and conditional entropy for a dyad performing a MT performance. **(B)** The mutual information of the same team. **(C)** The *MI* expression at different sensor sites. **(D)** *MI* expression at different frequencies.

The first example shows the dynamical relationships among these variables for a Map Task performance (Figure 6). The modeling was performed with the CzP0 sensor bipole and is shown for the 10 Hz frequency. The *JE* profile for this performance was not uniform but showed decreases between 60 and 130 s, 140 and 200 s, and a broad decrease between ~335 and 475 s. The profile of the *CE* showed larger decreases than the *NS*_*H*_ profile indicating the presence of shared 10 Hz information between the two persons. Figure 6B shows the dynamics of this shared information in the form of *MI*. *MI* is always a positive value and the *MI* profile was complementary to the difference between the *JE* and *CE* in Figure 6A. The *MI* accounted for ~2% of the *JE* when averaged over the entire performance, and during the 60 and 130 s period and 140 and 200 s periods the proportion was enriched to ~4 and 3% respectively.

A more global view of team neurodynamics is shown by plotting the *MI* expression over time as a function of the EEG sensor location (Figure 6C), or EEG frequency bin (Figure 6D). Mutual information was detected throughout most of the performance at some sensor sites with the highest average *MI* levels found in the Fz, C3, C4, CzP0, and F3 sensors. There was minimal *MI* in the 3–8 Hz frequency bins while the highest *MI* levels were found in the 14–17 Hz bins.

The MT example indicated that quantitative relationships existed in the *IE* data streams of dyads and that it might be possible to do similar modeling between the members of larger teams. The relative levels of *JE* and *CE* compared with *MI* also suggested the presence of noise in the overall modeling approach. The next two examples address this issue using other properties of information theory.

One useful property of information theory is that information is additive: the information associated with a set of outcomes can be obtained by adding the information of individual outcomes. We use this property in the following way: Our hypothesis was that the entropy levels of each person reflected his/her neurodynamic organizational responses to the other team members and the task (plus additional background noises in the brain). An individual with three possible EEG power states (i.e., high, medium, low) would have a maximum entropy of 1.585 bits. For a three person team each with three possible states, the maximum number of symbols that could be expressed is 27 and the maximum information is log_2_(27) or 4.755 bits. From the additive rule, the maximum information in three individual data streams should equal the information in a three person team i.e., (1.585 × 3 = 4.755 bits). This additive rule provides a basis for comparing the amounts of neurodynamic organization of each team member and the contributions of individual team members' organization to the overall team's neurodynamic organization.

As shown in Table 1 the average team entropy calculated when the team was modeled from the 27 symbol state space in Figure 2A was significantly lower than when the three *IE* levels were added together (*Mean*_*IndEnt*_ = 4.44 bits ± 0.18 vs. *Mean*_*TeamEnt*_ = 4.18 bits ± 0.045 (*SD), t* = 15.3, *df* = 4, *p* < 0.01).

**Table 1:** **Comparison of the entropy levels calculated by summing the IE of three team members or by directly modeling the *NS*_*H*_ using the 27 *NSS* in Figure 2A**.

**Team**	**Sum of IE**	***NS*_*H*_**
1	4.44	4.12
2	4.22	4.01
3	4.35	4.15
4	4.53	4.27
5	4.21	3.94
6	4.68	4.37
7	4.68	4.41
Mean	4.44	4.18

**Figure 7:**
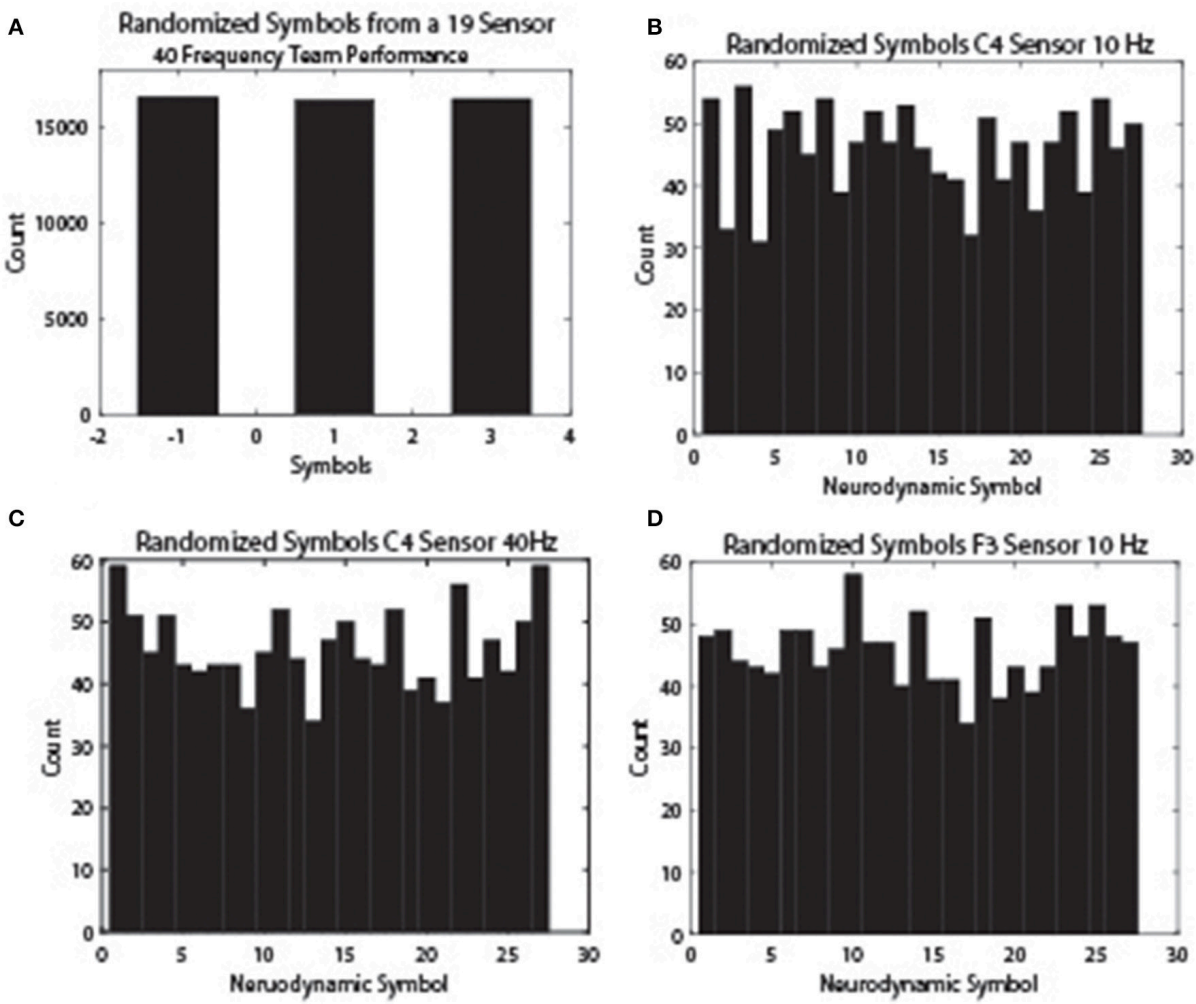
**NS distributions across EEG frequency and sensor sites**. The neurodynamic symbol streams were randomized to remove temporal structures and histogram plots were prepared to show the NS distributions. **(A)** The distribution of the −1, 1, and 3 symbol categories for a team performance. **(B)** The symbol distribution of the 10 Hz frequency band from the C4 sensor. **(C)** The symbol distribution of the 40 Hz frequency band from the C4 sensor. **(D)** The symbol distribution of the 10 Hz frequency band from the F3 sensor.

The reason for the difference is that the symbols in the *IE* data streams were divided equally into three groups and so the −1, 1, and 3 symbols were equally expressed. The 27 symbols in the *NS*_*H*_ were not similarly constrained and some symbols are repeated more frequently than others as part of the natural rhythm of the team on the task. This decreased variability differs on a frequency and sensor specific basis and results in a lower entropy levels. These relationships are shown in Figure 7 for the 10 Hz frequency bands from the C4 (Figure 7B) and F3 (Figure 7D) sensors and the 40 Hz frequency band from the C4 sensor (Figure 7C). As expected from the modeling protocol, the three-level normalized symbol stream had equal numbers of the −1, 1, and 3 symbols (Figure 7A), while the *NDS* from the different sensors and frequency bands showed variable symbol distributions.

The unequal symbol expressions seen after randomizing the *NDS* may indicate and important organization property of teams. One idea is that the task demands encourage/select particular neurodynamic relationships across the members of a team. To the extent these symbols are consistently associated across team activities or teams (novice/expert for instance) they may indicate important team member relationships relative to the task demands. We term the entropy associated with these symbol distributions the Task Entropy, or *H*_*Task*_. With these considerations, the sum of the individual entropy from the team members is used in the following sections when calculating the proportion of time team members are synchronized with each other using *MI*.

### Submarine navigation team

**Figure 8:**
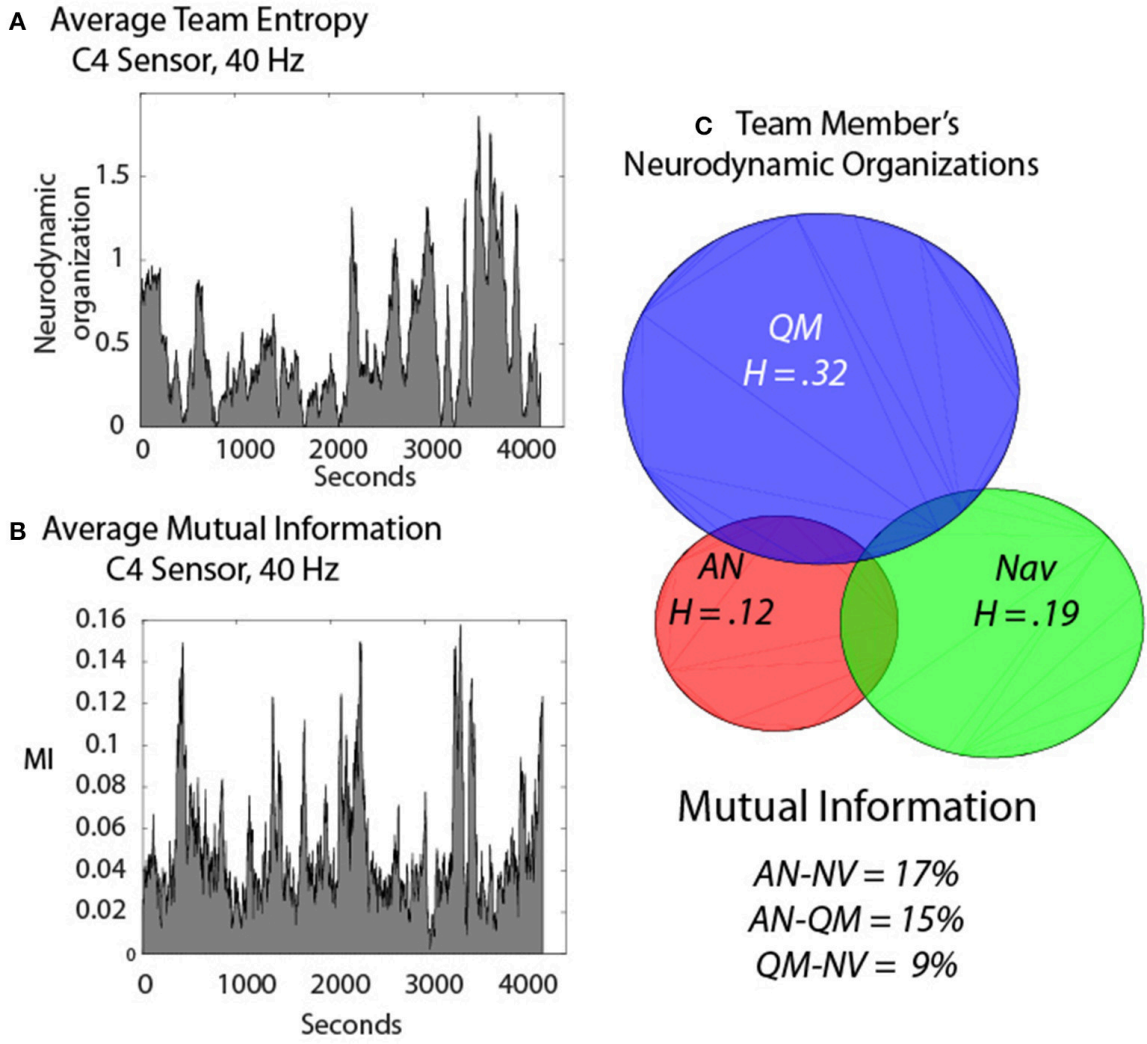
**Neurodynamics of a three-person submarine navigation team. (A)** The Neurodynamic Organization profile of the summed *IE* **(A)** or *MI* **(B)** from the Assistant Navigator (AN), the Quartermaster (QM) and the Navigator (NV) during a simulated navigation exercise. **(C)** Venn diagram of the individual IE levels and the degree of team synchrony determined by the *MI* of the dyads.

The next example was a three-person navigation team that performed a required submarine piloting and navigation simulation exercise. In an effort to remove unwanted noise from the modeling we subtracted the *IE* from the entropy of frequency and sensor-matched *IE* that had been randomized before the entropy calculations. We term the resulting value Neurodynamic Organization when applied in a team context, and abbreviate it *ND*_Ω_. This resulted in positive values that could be directly compared with *MI* (Figure 8); the dynamics of the *ND*_Ω_ and *MI* data streams are shown in Figures 8A,B.

Figure 8C shows the *ND*_Ω_ for the Assistant Navigator (ANav), the Quartermaster (QM), and the Navigator (Nav) and the circles are proportional to the overall levels of individual *ND*_Ω_. The overlap of the circles in the Venn diagram represents the levels of synchrony (as measured by *MI*) among the three team members, and the levels are labeled below. Removing the “noise” in the *NDS* by subtracting the *IE* of each person from randomized values of frequency and sensor matched *IE* streams resulted in higher proportions of *MI* being detected across the team members, as compared with the *MT* studies, being as high as 17% between ANav-NV when averaged across nearly 2 h. of teamwork.

### Healthcare teams

**Figure 9:**
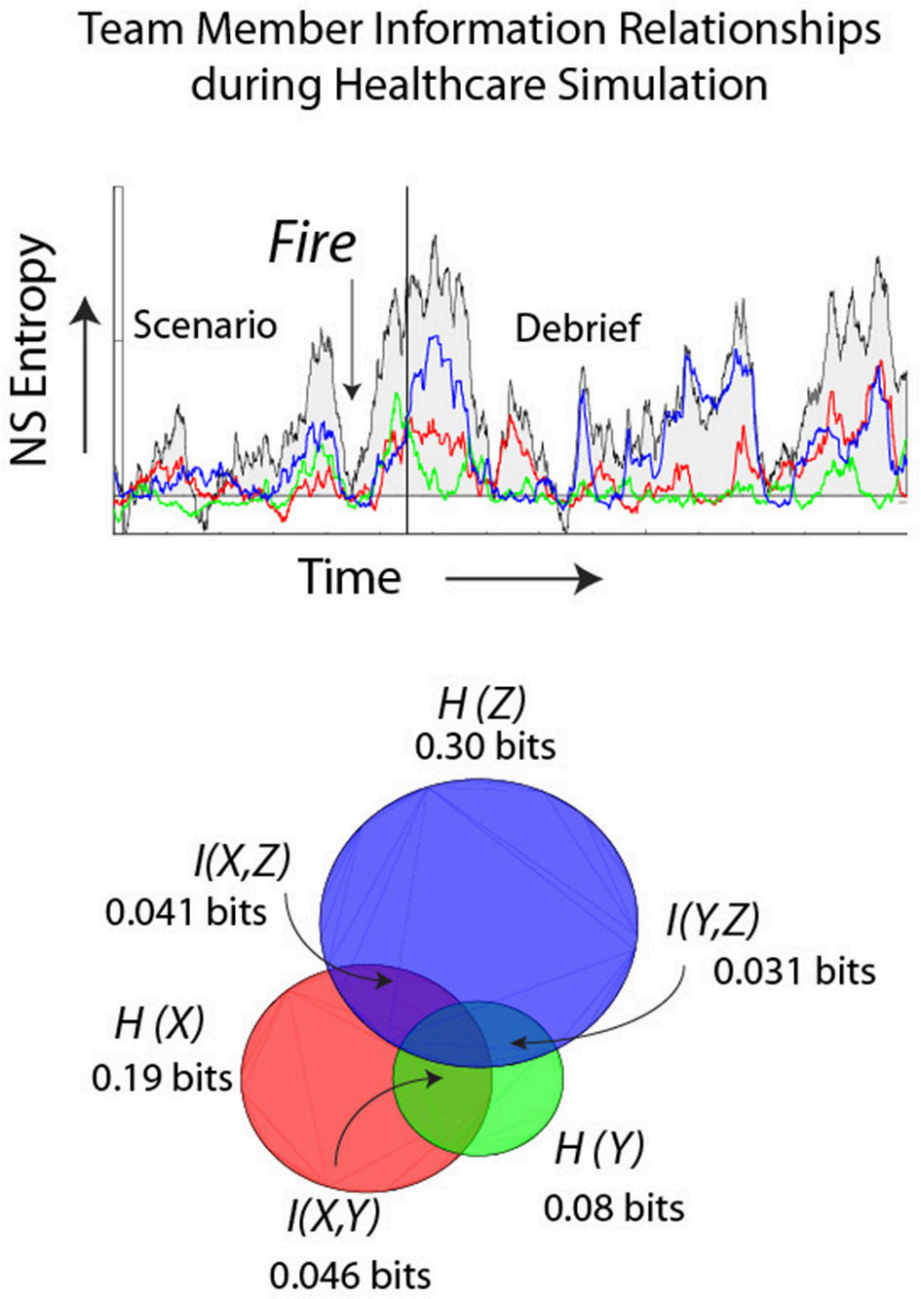
**Dynamics of a healthcare team during a simulated fire in the operating room**. The individual entropy levels were calculated using the normalized frequency-specific data symbols (i.e., −1, 1, 3) over a moving window of 60 s. The colored traces show the *IE* entropy remaining when the team members' entropy were subtracted from parallel frequency-matched entropy from data streams that were randomized before calculating entropy. The background trace is the team *ND*_Ω_. The lower Venn diagram shows the *IE* for the anesthesiologist (AN), registered nurse (RN) and the scrub tech nurse (ST). The *MI* of the dyad pairs are shown in the overlapping regions.

The final example extends these ideas by providing a more dynamical perspective of IE in relation to the *ND*_Ω_. The healthcare simulation illustrated in Figure 9 was designed to induce uncertainty/surprise in the team as it involved a patient undergoing an operation where shortly after anesthesia was induced the team had to evacuate the operating room with the patient due to a fire. The figure shows the *IE* traces for the anesthesiologist (red), scrub tech nurse (green), and a registered nurse (blue). The low *ND*_Ω_ just prior to the fire rose and continued to rise for each team member until the end of the simulation indicated by the solid line. As the team adjourned to the Debriefing room the *ND*_Ω_ returned to lower levels. The sum of the three team member's *IE* closely paralleled that of the team neurodynamic entropy (i.e., *NS*_*H*_) modeled from the 27 symbols in Figure 2A. The mutual information between the different dyad pairs is shown in the Venn diagram in relation to the summed *IE* levels of the three team members. The % of the individual entropy that was *M*I was highest, 62% for the AN and ST, 29% for the AN & RN, and 10% for the ST and RN.

## Discussion

In this study we have used information-organization concepts to develop metrics for the quantitative neurodynamic modeling of team performance. After beginning from a theoretical perspective of a perfect team, we highlighted team performances of three tasks that were very different in their content domains and team compositions.

The first example with high school dyads showed the relationships between the neurodynamic organizations as measured by *NS*_*H*_ and synchrony of the team as measured by *MI*. It further illustrated that while there was some redundancy in *MI* expression at different scalp positions, the different EEG sensor data provided different perspectives of the performance; similar ideas apply to the EEG frequency data as well.

The second example with a submarine navigation team introduced comparisons between individual *NS*_*H*_ and *MI* for the dyad combinations of a three-person team and provided quantitative estimates of the proportion of neurodynamic organization that might represent synchronization (as measured by *MI*) for each team member as well as the degree of (neurodynamic) interactions among the different team members. The third example expanded these ideas for a healthcare team and dynamically illustrated the changing *IE* relationships among the team members.

The innovative feature of this modeling process is the transformation of the physical units of the raw EEG power (in microvolts) from different team members into a single symbolic information stream where the symbols represent the relationships of the different team members with each other and with the second-by-second evolution of the task. At the junction of this transformation two modeling pathways result: (1) the raw EEG power levels that can be analyzed at scales of 2 s or less for dynamics that relate to mental imagery, social coordination, emotions, etc.; (2) and the entropy levels (bits of information) of the neurodynamic organizations in the symbol streams that relate more easily with other information measures like the organization of speech or the behavioral organizations recognized by experts as proficiency. This transformation provide a teamwork link analogous to the connection between thermodynamics and information theory in individuals detailed by Collell and Fauquet (2015).

The results to date suggest that higher performing teams are those characterized by more variability (i.e., higher entropy levels). The most interesting data streams to study though for understanding how to assemble, train and support teams might be those with less variability (i.e., lower entropy levels). These periods are often seen associated with stress or uncertainty, and when teams develop new neurodynamic organizations as they seek to acquire/synthesize additional information (Stevens et al., 2013, 2016a).

The similar findings with three different tasks suggests that the variables we are studying, and their resulting dynamics, may be a fundamental property of teams performing complex tasks. If so, this line of research has the potential to inform many practical applications related to team performance and resilience, as well as foster the development of new theoretical understandings about physiological synchronizations associated with social coordination and teamwork.

The principle driving this line of research is that teams adopt a more organized configuration, neurodynamically speaking, when seeking new/different information and organizations to balance the demands of the changing environment. When these challenges/uncertainties are resolved the team once again restructures to adopt a more efficient configuration; it may or may not be the same organization as before the perturbation. The length of these periods can be seconds, or much longer depending on the nature of the “surprise” experienced and the amount of new information that has to be acquired, synthesized, and exchanged before the team can return to a normal operating mode. These dynamics are consistent with the multifractal scaling in *NDS* previously seen in the neurodynamic data streams of submarine teams (Likens et al., 2014). The across sensors and frequencies *IE* and *MI* variability in Figures 6C,D may provide one explanation for the multifractal structures seen in those studies.

The three examples also hint at the dimensionality challenge of team neurodynamic modeling. Information theory is fundamentally about signals, not the meaning they carry; linkages to more human—understandable measures are needed to extract what the neurodynamic organizations/synchronizations “mean” to a team. This contributes to the dimensionality problem. As an example, with 19 EEG sensors and 40 (1 Hz) frequency bins, there are 760 sensor x frequency combinations per person to model over tasks lasting 500–4,000 s or more. The data streams include raw EEG data, data symbols, individual entropy, joint entropy conditional entropy, mutual information and team entropy, each of which has different properties/uses. Additionally, real-world, complex tasks often include segments with very different team requirements (i.e., Briefing, Scenario, Debriefing), along with shorter periods of organization relating to the momentary demands of the task. For validity and relevance, other measures are needed like speech flow, or speech content, instructor ratings and/or sub-dimensions of ratings like dialogue, problem solving, teamwork, etc. The additional measures may not always provide increased clarity. In a recent study the cross-level effects between the dynamics of communication and neurodynamics were modeled (Gorman et al., 2016). One interesting findings was a difference in the temporal lags between the neural and communication data streams between novices and experts, indicating that relating variables to each other at zero time lag may be insufficient to understand the interrelated system dynamics, and that changing time dimensions may also be needed during modeling.

More optimistically, the cross-couplings in that study also showed that redundancy exists between speech and neurodynamics. Similar redundancies are also seen between nearby EEG frequency bands, and also across EEG sensor sites, and so only a subset of the theoretical combinations of the above variables will be needed to encompass the major fundamental interactions among team members (Carandini and Heeger, 2012).

In several small-scale studies we have approached this modeling complexity by linking behavioral observations with neurodynamic organization measures (Stevens et al., 2015, 2016c, 2017). Currently, most evaluations of teams performing natural tasks rely on experts who observe and rate teams across important, but quantitatively vague dimensions like leadership, team structure, and situation monitoring using vetted rubrics. One widely used evaluation rubric in healthcare is the TeamSTEPPS® program which was developed by the Department of Defense for evaluating teams across dimensions that are prevalent in healthcare, but common to many professional teamwork situations (Baker et al., 2009). A more recent instrument, the Submarine Team Behavior Toolkit (STBT), focuses on team resilience and was designed for evaluating training and on-the-job teaming in the submarine force (described in Stevens et al., 2015). These scales tend to rely on macro features of team performance by summarizing observations over extended periods of time. While the shorter-term dynamics of the team are implicitly acknowledged in the resulting ratings, the dynamical details are often lost.

**Figure 10:**
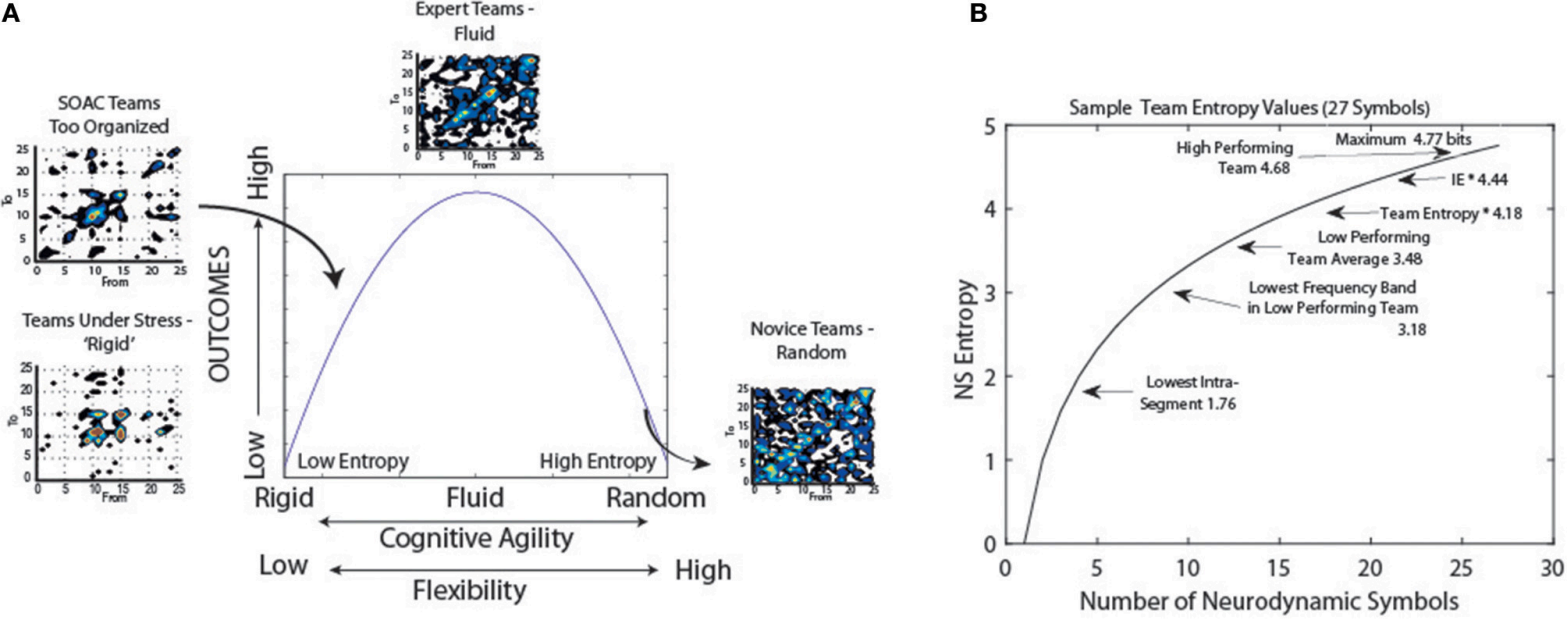
**Models of neurodynamic organizations**. **(A)** Prior neurodynamic organization model (Stevens et al., 2013). **(B)** Plot of the entropy levels as a function of the number of symbols. The labels position levels of different teamwork functions.

In an earlier study we proposed a bell-shaped relationship between what we then termed cognitive organization and team performance (Stevens et al., 2013). The cognitive organization was based on *NS*_*H*_ where the lower the entropy the more neurodynamically organized the team. These organizational/performance relationships were illustrated by plotting transition matrices of the *NDS* symbols at times *t* vs. *t*+*1 s*, and doing so for teams of different experience. Teams experiencing stressful situations showed the greatest degree of neurodynamic organization, followed by teams with some experience engaged in advanced training. At the other end of the curve were teams with little domain knowledge or experience; these were the least organized teams. Experienced teams were shown at the top of the curve, a balance of flexibility and organization (Figure 10A).

A restructured version of this model is shown in Figure 10B which more empirically encompasses our understandings of *NS*_*H*_ levels during teamwork. The asymptotic shape of the curve reflects the relationships between the information (*NS*_*H*_) and the number of symbols in the data stream. At the high end of the curve, some high performing teams have approached the theoretical maximum entropy levels while the lowest level of *NS*_*H*_ we have observed reflects a team using only 3–4 symbols of the 27-symbol *NSS*. This range provides a relatively broad range of the curve over which to use information measures to probe team dynamics and performance.

Expressed in terms of neurodynamic organizations (*ND*_Ω_), this would represent levels of 0 to ~ 1.5 bits. What does this bit or so of information tell us about the past, present, and future of the data stream? When thought of in terms of a single observation, if the sequence is a series of alternating 1's and 0's it tells us everything, while if the series contains random 1's and 0's it tells us very little (James et al., 2011). From the transition matrices in Figure 10A, one observation will likely tell us quite a lot. The existence of the diagonal in the *t* − > *t*+*1* transition matrix of Experienced Teams indicates that the *NDS* has long memory, the statistical dependence of two points with increasing time intervals, a property shared by many real-world data time series (Palva et al., 2013). Furthermore, the thickness of the diagonal tells us that the next symbol in a sequence may not be exactly the current one, but one closely related on the 27 symbol topological *NSS*. So short term-we learn a lot from a single observation. This will be particularly true for teams in training who have some experience and are refining their skills; the team in Figure 9A highlighted as having moderate organization. The challenge will be that the most novice teams will have very high levels of entropy and may be indistinguishable from noise, or more problematically, very experienced teams.

Finally, in the Introduction we posed the questions: (1) Can we begin to populate models of teamwork with quantitative data that reflects what is understood to be expertise? (2) Are teams in fact fully connected, and if so, how tightly linked are the couplings across different team members and different teamwork measures? (3) Are there preferred couplings among team members depending on the task, or training protocol, or training site, and does this make a difference? (4) How closely related are the models being revealed by neurodynamics, communication and behavioral measures. From the findings reported in the Results we feel these questions are all approachable.

One important relationship reported in this paper is that between the *IE* of the team members and the *MI* between dyad pairs of the team. For the first time it is possible to put quantitative relationships between the dynamics of each team member during the task, along with the neurodynamic interactions between the members of the team. While the three-person examples in Figures 8, 9 show the aggregated couplings among team members it is an easy extension to develop dynamic networks that show momentary relationships. These dynamical models enable comparisons with measures of team communication (Gorman et al., 2016) as well as behavioral models derived from expert raters (Stevens et al., 2015, 2016c), leading to dynamic multi-level, multi-modal and multi-entity snapshots of novice and expert teams in action.

We therefore see the further development of these methods (in particular, to consider the information provided by many spatial and temporal scales simultaneously), as an important area for developing the computational neuroscience of teams for some years. We also see increased opportunities to restructure team training. To the extent that neurodynamic organization equates to individuals and teams experiencing and resolving uncertainty (Stevens et al., 2016a) it may provide an indicator of where training should be focused.

## Author contributions

All authors listed, have made substantial, direct and intellectual contribution to the work, and approved it for publication. The authors jointly developed the design, performed the neurodynamic analyses, and wrote the paper.

### Conflict of interest statement

The authors declare that the research was conducted in the absence of any commercial or financial relationships that could be construed as a potential conflict of interest. The reviewer TK and handling Editor declared their shared affiliation, and the handling Editor states that the process nevertheless met the standards of a fair and objective review.
